# An Evaluation of Intranasal Sufentanil and Dexmedetomidine for Pediatric Dental Sedation

**DOI:** 10.3390/pharmaceutics6010175

**Published:** 2014-03-21

**Authors:** James M. Hitt, Toby Corcoran, Kelly Michienzi, Paul Creighton, Christopher Heard

**Affiliations:** 1 Department of Anesthesiology, University at Buffalo, Buffalo, NY 14260, USA; 2 Department of Community and Pediatric Dentistry, State University of New York at Buffalo, Women and Children’s Hospital, Buffalo, NY 14222, USA; E-Mails: Tobycorcorandds@gmail.com (T.C.); kmichienzi@kaleidahealth.org (K.M.); pcreighto@gmail.com (P.C.); 3 Department of Pediatric Anesthesiology, Division of Pediatric Critical Care, State University of New York at Buffalo, Women and Children’s Hospital, Buffalo, NY 14222, USA; E-Mail: heardop1@verizon.net

**Keywords:** pediatric sedation, pediatric dentistry, intranasal drug administration

## Abstract

Conscious or moderate sedation is routinely used to facilitate the dental care of the pre- or un-cooperative child. Dexmedetomidine (DEX) has little respiratory depressant effect, possibly making it a safer option when used as an adjunct to either opioids or benzodiazepines. Unlike intranasal (IN) midazolam, IN application of DEX and sufentanil (SUF) does not appear to cause much discomfort. Further, although DEX lacks respiratory depressive effects, it is an α_2_-agonist that can cause hypotension and bradycardia when given in high doses or during prolonged periods of administration. The aim of this feasibility study was to prospectively assess IN DEX/SUF as a potential sedation regimen for pediatric dental procedures. After IRB approval and informed consent, children (aged 3–7 years; *n* = 20) from our dental clinic were recruited. All patients received 2 μg/kg (max 40 μg) of IN DEX 45 min before the procedure, followed 30 min later by 1 μg/kg (max 20 μg) of IN SUF. An independent observer rated the effects of sedation using the Ohio State University Behavior Rating Scale (OSUBRS) and University of Michigan Sedation Scale (UMSS). The dentist and the parent also assessed the efficacy of sedation. Dental procedures were well tolerated and none were aborted. The mean OSUBRS procedure score was 2.1, the UMSS procedure score was 1.6, and all scores returned to baseline after the procedure. The average dentist rated quality of sedation was 7.6 across the 20 subjects. After discharge, parents reported one child with prolonged drowsiness and one child who vomited at home. The use of IN DEX supplemented with IN SUF provided both an effective and tolerable form of moderate sedation. Although onset and recovery are slower than with oral (PO) midazolam and transmucosal fentanyl, the quality of the sedation may be better with less risk of respiratory depression. Results from this preliminary study showed no major complications from IN delivery of these agents.

## 1. Introduction

Conscious or moderate sedation is routinely used to facilitate the dental care of the pre-cooperative or uncooperative child [1,2,3,4]. Children who fail moderate sedation often need general anesthesia in order to complete their dental procedure, adding, cost, risk, and a delay of care.

Intranasal administration is a novel way to administer CNS-active compounds, circumventing the issues related to enteral absorption and first-pass hepatic metabolism. This is of particular interest in the pediatric population because the IV route of administration can be difficult for the practitioner and cause undue stress in the patient, and the IN route of administration presents a good alternative in the pediatric population [5]. The blood–brain barrier (BBB) limits the penetration of medications to the brain via multiple mechanisms [6], and intranasal drug delivery circumvents typical BBB obstacles by penetrating the cribriform plate and potentially utilizing paracellular, transcellular, or active neuronal transport mechanisms [7].

At our dental clinic we provide dental sedation using either oral (PO) or intranasal (IN) medications [8]. Current options include oral (PO) or intranasal (IN) midazolam (MID), IN sufentanil (SUF), or IN MID with oral transmucosal fentanyl citrate (OTFC). Oral midazolam alone can result in insufficient sedation to allow the dental procedure, and the addition of an opioid may increase the risk of respiratory depression or vomiting [2].

Dexmedetomidine is a selective α_2_-agonist that causes sedation with minimal respiratory depression [9], making it a safer option for sedation when used in conjunction with opioids or benzodiazepines. Dexmedetomidine has been used intravenously in an intensive care unit (ICU) [9] and for procedural sedation such as magnetic resonance imaging (MRI) [10]. The pharmacokinetics of dexmedetomidine intravenous (IV) infusions in children has shown a terminal half-life of 1.8 h [11]. Intravenous infusions of dexmedetomidine require the initial use of a loading dose, and higher doses of dexmedetomidine have been associated with a delay in recovery [12]. A single, slower-acting intranasal dose without continued infusion or IV bolus may reduce the risks for any blood pressure or heart rates issues that can occur with IV dexmedetomidine.

There have been several case reports on the use of IV dexmedetomidine for sedation for radiographic investigations [10,13,14,15,16,17], and a recent study reported that IN DEX was comparable to IN MID in producing pre-procedural sedation for children undergoing general anesthesia for complete dental rehabilitation [18]. In addition, while intranasal administration of midazolam is associated with some discomfort [18,19], intranasal dexmedetomidine and sufentanil cause minimal discomfort on administration [10,18,20,21,22]. This prospective, non-comparative study was designed to assess the feasibility IN DEX/SUF sedation in pediatric patients undergoing dental procedures.

## 2. Methods

Prior to the study, IRB approval was obtained from the Children and Youth Institutional Review Board at Woman and Children’s Hospital of Buffalo. Twenty patients, ages 3–7 years, scheduled for moderate sedation were recruited. The children underwent a combination of dental restorations, stainless steel/strip crowns, and extractions. All children did not tolerate their procedure with nitrous oxide analgesia and were otherwise candidates for general anesthesia in order to complete their dental procedures. Exclusion criteria were: refusal or inability to get informed consent, age <3 or >7 years, body mass index (BMI) <5% or >95%, allergy to synthetic opioids, or allergy to clonidine or dexmedetomidine. 

After informed consent was obtained parents and children were interviewed and examined in the pre-procedure holding area, and the sedation protocol was initiated 45 min prior to the procedure. All sedation medications were administered by a pediatric anesthesiologist, intra-nasally using the Mucosal Atomization Device (MAD^®^ Nasal, Wolfe Tory Medical, Inc., Salt Lake City, UT, USA). All patients received intranasal dexmedetomidine (IN DEX) 2 μg/kg (max dose 40 μg), followed in 30 min by intranasal sufentanil (IN SUF) 1 μg /kg (max dose 20 μg) to the contralateral nares. An independent observer (research nurse) scored the discomfort associated with IN drug administration on a scale of 1–10 (no pain to very painful). Children were observed in the pre-procedure holding area by their parents and the clinic nurses. Forty-five min after the dose of IN DEX, the patient was transported to the procedure room and attached to electrocardiogram, noninvasive arterial blood pressure, and pulse oximeter monitors. A single observer not performing the procedure, recorded any episodes of bradycardia (heart rate < 50), respiratory depression, obstruction or desaturation.

During the procedure, the independent observer recorded the child’s behavior using the OSUBRS (see Table 1) [23] and the UMSS (see Table 2) [24]. In addition, the dentist and dental resident subjectively rated the quality of sedation on a scale of 1–10 (poor to excellent) at the conclusion of the procedure. 

During the procedure and in the post-anesthesia care unit (PACU), children were assessed for any side effects and complications of the sedation. This included excessive post-procedural sedation (PACU time >90 min or room air pulse oximetry (SpO_2_) <94%), hemodynamic changes requiring intervention, and nausea or vomiting. Children were discharged from the PACU after they met the following criteria: Time in recovery >20 min, returned to baseline mental status, hemodynamically stable, SpO_2_ > 95%, ambulatory, and not complaining of nausea or pain.

Parents were contacted by phone, within 48 h of the procedure to inquire about adverse events after discharge and to assess their satisfaction with the effects of the sedation administered to their child. 

**Table 1:** Ohio State University behavior rating score.

Score	Behavior
1	Quiet behavior, no movement
2	Crying, no struggling
3	Struggling movement without crying
4	Struggling movement with crying

**Table 2:** University of Michigan sedation score.

Score	Degree of sedation
0	Awake and alert
1	Minimally sedated
2	Moderately sedated
3	Deeply sedated but rouseable
4	Unarousable

## 3. Results

Twenty children completed this study. Demographic data are presented in Table 3. The mean age was 4.5 ± 1.0 years and mean weight was 18.2 ± 4.2 kg. The mean sedation time was 46 ± 5 min, and the mean procedure time was 50 ± 18 min (Table 4). The mean IN DEX dose was 34.9 μg and the mean IN SUF dose was 17.4 μg, which were consistent with our previous experience. The intranasal administration of medications was successful in all 20 children enrolled in the study. Observed discomfort on injection was 2.6 ± 1.8 for dexmedetomidine and 2.8 ± 2.1 for sufentanil as rated by an independent observer on a 10-point scale. 

**Figure 1:**
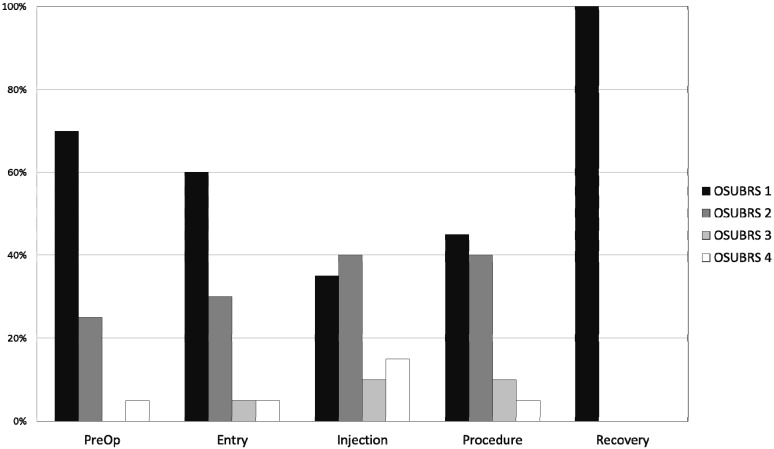
Histogram plot of Ohio State University Behavior Rating Scale (OSUBRS) values (see Table 1) at various time points: pre-procedure hold area; entry to the procedure room; injection of local anesthetic; during the procedure; and 5 min after admission to the post-anesthesia care unit (PACU).

**Figure 2:**
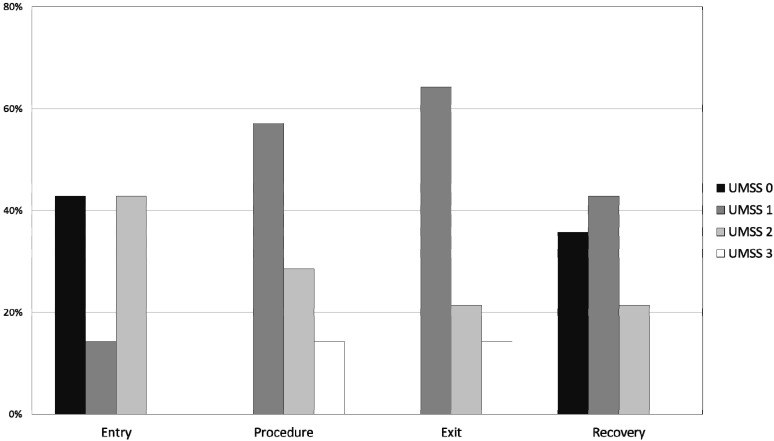
Histogram plot of University of Michigan Sedation Scale (UMSS) values (see Table 2) at different time points: entry to the procedure room; during the procedure, immediately prior to leaving the procedure room; and 5 min after admission to the PACU.

All children tolerated the dental procedure without major difficulty and no procedures were aborted due to noncompliance. The OSUBRS deteriorated slightly during the procedure (Table 5), increasing from 1.6 ± 0.8 on entry to the room to 2.1 ± 1.1 at the start of the procedure. Figure 1 shows the percentage distribution of each OSUBRS at various time points of assessment. Cooperation deteriorated from the pre-procedural holding area to the local anesthetic injection (increase OSUBRS scores; see Figure 1), but all procedures were completed in the office setting. Satisfactory sedation was achieved in all 20 patients, as demonstrated by no UMSS equal to 0 during the procedure, and there were no instances of oversedation (UMSS = 4; see Table 5). The degree of sedation increased from the pre-sedation baseline score of 0, but the UMSS values (Figure 2) indicated that all of the children were rated as being between minimally and moderately sedated. The dental residents (mean 7.8 ± 2.4) and dental attending (mean 7.6 ± 2.4) both rated the procedural sedation highly using the 1 to 10 rating score.

All planned dental cases were completed during the study, and no cases were interrupted due to side effects of the sedation regimen. One episode of airway obstruction was encountered during the procedures, and it was addressed with conservative measures (*i.e.*, neck extension and jaw lift). There were no occurrences of airway obstruction in the PACU. There were no desaturation events either during the procedure or in the PACU, and there were no episodes of excessive sedation. There were no hyper- or hypo-tensive episodes either during the procedure or in the PACU. Recovery room discharge time averaged 55 min (Table 4), and the maximum time spent in PACU was 90 min. 

A parent was successfully contacted with a follow-up phone call for all 20 participants. Overall the parents were very satisfied with this sedation regimen. The mean parental satisfaction rating was a score of 8.8 ± 1.3 on a scale of 1 to 10, and 85% of the ratings were greater than 7. Two parent assessments of 7 related to perceived inadequacies of the clinic waiting room facilities. One parent rated their satisfaction at 5 out of 10 because of post-operative vomiting encountered at home that resolved without the need for any medical intervention. Thirty five percent of the parents reported that the children were drowsy at home, and one parent noted excessive drowsiness lasting 4 h after the procedure. 

**Table 3:** Demographics (*N* = 20).

Weight (kg)	18.2 ± 4.2
Age (years)	4.5 ± 1.0
DEX dose (μg)	34.9 ± 5.0
SUF dose (μg)	17.4 ± 2.5

**Table 4:** Study times.

Times	[min, (mean ± SD)]
Sedation time	46 ± 5
Procedure time	50 ± 18
Discharge time	55 ± 21
DEX-SUF time	30 ± 1
Total time	136 ± 24

**Table 5:** Behavior and sedation scores (mean ± SD).

Scale	Enter Room	Procedure	Exit Room	PACU
OSUBRS	1.6 ± 0.8	2.1 ± 1.1	1.8 ± 0.9	1.0 ± 0.0
UMSS	1.0 ± 1.0	1.6 ± 0.8	1.5 ± 0.8	0.9 ± 0.8

## 4. Discussion

Providing dental care to the pre-cooperative or uncooperative child can be a challenge, and failure to accomplish the procedure in the dental clinic can necessitate completing the procedure under general anesthesia, which is more costly, risky, and time consuming. Oral or intranasal midazolam has been commonly used for sedation in this situation, but in difficult cases it may not be sufficient. Failure rates of moderate sedation with a variety of agents (oral midazolam, IN midazolam, OTFC and IN midazolam, and IN midazolam with IN sufentanil) have been reported to range from 17% to 36% [12,25]. While our study of 20 children did not find any sedation failures, a larger scale study will be required to fully demonstrate efficacy and safety of this sedation regimen.

Oral midazolam is often well tolerated and produces acceptable sedation, but some children require additional medications for sedation. Opioids are commonly used, but when combined with benzodiazepines, there is an increased risk for respiratory depression. Dexmedetomidine is a selective α_2_-selective agonist with good bioavailability [26] when delivered by buccal or nasal mucosa. Intranasal dexmedetomidine is associated with an onset of sedation at 45 to 60 min after administration, with the peak sedation effect occurring at 90 to 105 min [22]. This delay in the onset of sedation was factored into our study protocol, with the dexmedetomidine being administered 45 min prior to the procedure start time. A local evaluation of the authors experience revealed that IN dexmedetomidine alone did not produce sufficient sedation and analgesia (Women and Children’s Hospital of Buffalo Pharmaceutical and Therapeutics Committee evaluation), so we sought to study the combination of DEX with an opioid. The combination of dexmedetomidine with a potent opioid offers the potential for increased efficacy of sedation with only one agent that inhibits respiration. 

Intranasal delivery of various opioids has been studied. Sufentanil is twice as lipophilic as fentanyl, it is rapidly absorbed from the nasal mucosa with excellent bioavailability of 78%, and IN sufentanil administration has been associated with stable hemodynamics, vital signs, and arterial oxygen tension [5]. Care must be taken when administering potent opioids to pediatric patients, in order to avoid overdose, especially respiratory depression [25]; although, in the event of opioid overdose, naloxone can be easily and effectively administered by the intranasal route to reverse the effects of the sufentanil [27]. We used a dose of 1 μg/kg because this dose has been previously reported as a safe and efficacious dose for sedation with no complications [6], and we used a maximum dose limit of 20 μg to increase the margin of safety. The timing of the sufentanil was based upon our previous experience of IN SUF with IN MID which had an onset time of about 15 min [8]. Even with the time allowed for the sedation to establish, the onset of our combination may in fact be even slower. In 43% of the children the UMSS on entry into the procedure was 0, and the UMSS increased in these patients over the following 5 to 10 min to one or greater. It may be possible to improve the quality of the sedation by waiting for signs of sedation in the pre-procedure area before transporting the patient to the procedure room.

The data from this feasibility study demonstrate that IN DEX and IN SUF is a viable sedation regimen for dental procedures lasting about one hour, given that all of the enrolled children completed their procedure, and the dentists were satisfied with the sedation provided by this regimen. The UMSS is a validated tool for studying pediatric sedation [24], but the measurements provide instantaneous measurements of sedation at select time points of the procedure rather than a continuous measurement of sedation during the course of the procedure. Because of this, the dentist evaluated the quality of the sedation, and, on average, rated the quality of sedation at 7.6 ± 2.4 on a 10-point scale.

The duration of the sedation allowed completion of procedures that lasted 50 min on average. This is also significantly longer than our experience from a variety of different sedation techniques, where we have found the time limit of procedural sedation to be often limited to 20 min [8]. The combination of IN DEX and IN SUF may allow dental procedures to be completed on one visit rather than multiple days, with obvious benefits in both over-all cost and convenience for the child and family.

We observed no serious episodes of airway obstruction or desaturation as a result of the sedation regimen during the observation period. After sedation, children were carefully watched and assessed prior to discharge home. Children were discharged home after 20 min when they were ambulatory, maintaining SpO_2_ on room air, and hemodynamically stable. The use of IN dexmedetomidine and sufentanil resulted in PACU observation times of 55 ± 21 min. This discharge time is longer than we have observed with our standard sedation regimens, typically consisting of benzodiazepines with or without opioids [8]. No child was nauseated during the study period, but one parent reported nausea in the 24 h after the procedure and sedation, further studies will be required to quantify the true incidence of post-procedural nausea associated with co-administration of IN DEX and IN SUF.

## 5. Conclusions

Intranasal delivery provides advantages over oral administration, and intranasal delivery of dexmedetomidine and sufentanil was tolerated by all 20 children in our study and resulted in acceptable sedation for procedures lasting about one hour. A comparative study of IN DEX/IN SUF and a regularly used sedation regimen, such as oral midazolam with or without opioid, is needed to directly compare the effectiveness of sedation and occurrence of adverse events with this new intranasal sedation combination.
